# Supplementary material for: Psychosocial and professional burden of Medically Assisted Reproduction (MAR): Results from a French survey
Source: PLoS One. 2020 Sep 24;15(9):e0238945. doi: 10.1371/journal.pone.0238945 (PMC7514013; doi:10.1371/journal.pone.0238945)
Supplement: S1 File — (DOCX) [file pone.0238945.s001.docx]

**Partie 1: Questions démographiques**

**A tous**

**RS1. Vous êtes :**

1. Un homme
2. Une femme

**A tous**

**RS2. Quelle est votre date de naissance ?**

**I__I__I Mois**

**I__I__I__I__I année**

1. **STOP INTER si <18 ans ou > à 50 ans**

**A tous**

**Q0. Quelle note entre 1 et 10 donneriez-vous aujourd’hui pour évaluer votre niveau de bien être en général ?** *1 signifie que vous ne vous sentez pas bien et 10 que vous vous sentez très bien. Les notes intermédiaires servent à nuancer votre jugement.*

1. Niveau de bien être général : /__/__/ / 10

**Aux personnes ayant des enfants**

**RS3. Parmi vos enfants, en avez-vous eu certains en ayant recours à une procréation médicalement assistée (PMA), également appelée assistance médicale à la procréation (AMP) ?**

Pour rappel une PMA ou une AMP est un ensemble de pratiques cliniques et biologiques où la médecine intervient plus ou moins directement dans la procréation pour concevoir un enfant.

1. Oui
2. Non 🡺 **STOP INTER**

**A tous**

**RS4. Quelle est votre situation de famille ?**

1. Célibataire
2. En union libre
3. Marié(e)
4. Séparé(e)
5. Divorcé(e)
6. Veuf/ve
7. Pacsé(e) (Pacte civil de solidarité).

**Aux hommes en union libre, mariés, séparés, divorcés ou pacsés**

**RS5. Avez-vous recours actuellement ou avez-vous eu recours à une procréation médicalement assistée (PMA), également appelée assistance médicale à la procréation (AMP) ?**

1. Oui
2. Non **🡺 STOP INTER**

**Aux femmes**

**RS6. Etes-vous enceinte ?**

1. Oui
2. Non

**Aux femmes enceintes**

**RS7. Avez-vous été enceinte après une procréation médicalement assistée (PMA), également appelée assistance médicale à la procréation (AMP) ?**

1. Oui
2. Non

**Aux personnes sans enfants :**

**RS8. Avez-vous recours actuellement ou avez-vous déjà eu recours au cours de votre vie à une procréation médicalement assistée (PMA) également appelée assistance médicale à la procréation (AMP) dans le but de concevoir un enfant ?**

1. Oui
2. Non 🡺 **STOP INTER**

**A tous**

**RS9. Aujourd’hui où en êtes-vous dans votre parcours de PMA ou AMP ?**

1. Je suis actuellement en parcours de PMA/AMP en France
2. Je suis actuellement en parcours de PMA/AMP à l’étranger **🡺 STOP INTER**
3. J’ai terminé le parcours car je suis parent grâce à une PMA/AMP réalisée à l’étranger **🡺 STOP INTER**
4. J’ai terminé le parcours car j’attends un enfant grâce à une PMA/AMP réalisée à l’étranger **🡺 STOP INTER**
5. J’ai terminé le parcours de PMA/AMP car je suis parent
6. J’ai terminé le parcours de PMA/AMP car j’attends un enfant
7. J’ai arrêté le parcours de PMA/AMP par choix personnel
8. J’ai arrêté le parcours de PMA/AMP car il n’est plus pris en charge par la sécurité sociale
9. J’ai arrêté le parcours de PMA/AMP car les médecins ne veulent plus me prendre en charge pour des raisons médicales
10. J’ai arrêté le parcours de PMA/AMP à cause d’un trop grand nombre d’échecs
11. Je fais une pause dans le parcours de PMA/AMP
12. Je suis en attente pour débuter un protocole de PMA/AMP car je suis sur file d’attente

**Si actuellement en parcours (RS9=1)**

**RS10. Dans quel type de protocole êtes-vous dans le cadre de votre parcours de PMA/AMP:**

1. Insémination artificielle avec sperme du conjoint (IAC)
2. Insémination artificielle avec don de sperme (IAD)
3. Fécondation *in Vitro*
4. Fécondation *in Vitro* avec don de gamètes (ovocytes/spermatozoïdes)
5. Accueil d’embryon(s)
6. Situations avec transfert d’embryon (TEC ou TEV, accueil d’embryon)

**A tous**

**RS11. Combien de parcours de PMA/AMP avez-vous effectué au cours de votre vie ?**

*Par parcours d’AMP nous entendons l’entrée dans un procole d’AMP pour lequel vous avez été suivi(e) par un médecin spécialisé en AMP et au cours duquel vous avez pu bénéficier de différents types d’aides médicales comme les inséminations artificielles et la FIV jusqu’à ce que vous ou le médecin décide d’arrêter l’AMP ou que vous soyez enceinte.*

I__I__I parcours AMP

**A tous**

**RS11. Et combien de tentatives avez-vous réalisées dans le cadre de votre/vos parcours de PMA/AMP ?**

I__I__I tentatives

**Partie 2: Parcours avant l’AMP**

**A tous**

**Q1. Parmi les suivants, quels ont été les éléments à l’origine de votre réflexion sur le recours à une AMP/PMA ?**

1. Suite à une consultation avec votre médecin généraliste
2. Suite à une consultation avec votre gynécologue
3. Une discussion avec des personnes de votre entourage
4. Des informations que vous avez trouvées sur internet
5. Des blogs, forums sur l’aide médicale à la procréation
6. Des campagnes d’information à la télévision, la radio, sur internet
7. Plusieurs mois ou plusieurs années sans réussir à concevoir un enfant

**A tous**

**Q2. Depuis quand essayez-vous d’avoir un enfant ?**

**I__I__I Mois**

**I__I__I__I__I année**

**A tous**

**Q3. Lorsque vous avez rencontré ces premières difficultés, quelles sont toutes les questions que vous vous êtes posées à ce moment-là ?**

1. Est-ce que c’est de ma faute ?
2. Est-ce que c’est de la faute de mon conjoint/compagnon ?
3. Est-ce que c’est héréditaire ?
4. Est-ce qu’il y a des antécédents dans ma famille ou celle de mon conjoint ?
5. Est-ce que je vais devoir adopter ?
6. Est-ce que je n’aurai jamais d’enfant ?
7. Est-ce que j’aurai le droit de bénéficier d’une AMP ?
8. Est-ce que c’est lié à mon alimentation ?
9. Est-ce que c’est lié à mon poids ?
10. Est-ce que c’est lié à mon mode de vie (tabac, pratique d’une activité physique, alcool...)
11. Est-ce que c’est lié à mon environnement professionnel ?

**A tous
Q4. Qui avez-vous rencontré en premier pour parler de vos difficultés à concevoir un enfant ?**

1. Le gynécologue qui vous suit/suit votre conjointe/compagne habituellement
2. Un médecin spécialisé en AMP
3. Votre médecin généraliste
4. Un endocrinologue
5. Une sage-femme
6. Un membre de ma famille/mon entourage (mère, soeur, ami(e))
7. Autre: Précisez : ____________________________

**A tous
Q5. Qui a posé le diagnostic de vos difficultés à concevoir un enfant ?**

1. Le gynécologue qui vous suit habituellement
2. Un gynécologue spécialisé en AMP
3. Votre médecin généraliste

**A tous**

**Q11. A quel moment avez-vous consulté par rapport à votre projet d’enfant ?**

1. Après moins de 6 mois d’essai
2. Entre 6 mois et 1 an d’essai
3. Entre 1 an et 18 mois d’essai
4. Entre 18 mois et 24 mois d’essai
5. Après 24 mois d’essai

**A tous**

**Q8. Quand a eu lieu la première consultation avec le médecin spécialiste en AMP ?**

**I__I__I Mois**

**I__I__I__I__I année**

**A tous**

**Q10. Parmi les sentiments suivants, quels sont les 3 que vous avez ressentis lors de la première consultation avec le médecin spécialiste en AMP ?**

1. Perdu (e)
2. Rassuré (e)
3. Espoir
4. Seul (e)
5. Angoissé (e)
6. Confiant (e)
7. Différent (e)
8. Honteux (euse)
9. En colère
10. Pris (e) en charge
11. Découragé(e)
12. Résigné (e)

**A tous**

**Q12. Dans le cadre de votre parcours AMP, quelles conseils ou recommandations vous a-t-on proposés lorsque vous avez eu le diagnostic de vos difficultés à concevoir un enfant ?**

1. Un changement d’alimentation
2. La pratique d’une activité physique régulière
3. Perdre du poids
4. Arrêter/diminuer votre consommation de tabac
5. Arrêter/diminuer votre consommation de canabis
6. Arrêter/diminuer votre consommation d’alcool
7. Une intervention chirurgicale

Un bilan d’infertilité complet du couple en vue d’une prise en charge rapide et active en PMA

Rien, juste continuer d’essayer et revenir dans quelques temps

Autre : Précisez : ___________________

**Partie 3: Parcours d’AMP**

*Introduction : nous allons maintenant revenir sur le parcours d’AMP que vous êtes en train de suivre ou que vous avez déjà suivi au cours de votre vie. Si jamais vous avez suivi plusieurs parcours d’AMP nous vous demandons de répondre aux questions suivantes en considérant le dernier parcours AMP/PMA pour lequel vous avez été suivi(e) par un médecin spécialisé en AMP et au cours duquel vous avez pu bénéficier de différents types d’aides médicales comme les inséminations artificielles et la FIV jusqu’à ce que vous ou le médecin décide d’arrêter l’AMP ou que vous soyez enceinte.*

**Si actuellement en parcours ou qui ont arrêté le parcours sans avoir eu d’enfant**

**Q13. Aujourd’hui, à quelle fréquence pensez-vous à votre désir d’avoir des enfants ?**

1. En permanence
2. Plusieurs fois par jour
3. Au moins une fois par jour
4. Plusieurs fois par semaine
5. Au moins une fois par semaine
6. Au moins une fois par mois
7. Moins souvent
8. Jamais

**A tous**

**Q14. Quand a-eu-lieu votre première consultation d’AMP ?**

**I__I__I Mois**

**I__I__I__I__I année**

**A tous**

**Q15. Quels sont les principaux sentiments que vous avez ressentis lors de votre première consultation d’AMP ?**

1. Perdu (e)
2. Rassuré (e)
3. Espoir
4. Seul (e)
5. Angoissé (e)
6. Confiant (e)
7. Différent (e)
8. Honteux (euse)
9. En colère
10. Pris (e) en charge
11. Découragé(e)
12. Résigné (e)

**A tous**

**Q16. En moyenne combien de temps vous faut-il pour aller à votre centre de PMA quel que soit le mode de transport que vous utilisez :**

1. Moins de 15 min
2. Entre 15 et 30 min
3. Entre 30 min et 1h
4. Entre 1h et 2h
5. Plus de 2h

**A tous**

**Q17. Diriez-vous que vous avez été très, plutôt, plutôt pas ou pas du tout satisfait (e) des éléments suivants dans le cadre de votre parcours d’AMP :**

1. Très satisfait (e)
2. Plutôt satisfait (e)
3. Plutôt pas satisfait (e)
4. Pas du tout satisfait (e)
5. L’accueil général (des personnels, des locaux...) lors de l’entrée dans le protocole d’AMP
6. Des explications fournies par le personnel médical (sur le parcours d’AMP, sur les raisons de vos difficultés à avoir un enfant...)
7. De la qualité des soins réalisés au centre d’AMP
8. De l’implication du personnel médical dans votre parcours d’AMP
9. De la disponibilité du personnel médical pour répondre à vos questions au cours du protocole de soins
10. Des aides/soutiens proposés (psychologue, associations de patients...) pour vous accompagner lors de votre parcours
11. De la durée moyenne de consultation dans le cadre de votre parcours d’AMP
12. De la capacité du personnel médical à répondre à vos questions
13. De la capacité du personnel médical à vous rassurer

**A tous**

**Q18. Dans le cadre de votre parcours d’AMP à quelle fréquence allez-vous sur les forums, réseaux sociaux...?**

1. Très souvent

2. Souvent

3. Rarement

4. Jamais

**A ceux qui vont sur les réseaux sociaux**

**Q19. Pour quelles raisons vous rendez vous sur les forums, les réseaux sociaux/forums dans le cadre de votre parcours d’AMP ?**

1. Pour vous renseigner sur les différentes étapes de l’AMP

2. Pour partager votre expérience

3. Pour avoir du soutien tout au long du processus, pour chercher du réconfort

4. Pour échanger avec des personnes qui vous comprennent

5. Pour vous exprimer librement

6. Pour témoigner de votre histoire

7. Pour dire ce que vous ne pouvez pas dire à votre conjoint/compagnon, à votre entourage ou au personnel médical de l’AMP

8. Pour trouver des réponses aux questions que vous vous posez sur votre parcours personnel

**Q20. Avez-vous déjà sollicité une ou de plusieurs association(s) ?**

1. Oui
2. Non

**A tous**

**Q21. Indiquez pour chacun des adjectifs suivants s’il s’applique oui ou non à la manière dont vous avez perçu le bilan diagnostic réalisé au début de votre parcours AMP :**

1. Oui
2. Non
3. Contraignant
4. Utile
5. Rassurant
6. Nécessaire
7. Déroutant
8. Long
9. Incompréhensible
10. Angoissant

**Aux personnes actuellement ou anciennement sous stimulation ovarienne par injection**

Q21BIS. Dans le cadre de votre traitement de stimulation ovarienne par injection, diriez-vous que vous êtes d’accord ou pas avec chacune des affirmations suivantes :

1. Tout à fait d’accord
2. Plutôt d’accord
3. Plutôt pas d’accord
4. Pas du tout d’accord
5. Non concerné

J’ai bien bien compris le traitement prescrit

Je me sens à l’aise avec les auto-injections

Je fais appel à une infirmière ou à mon entourage pour les injections

J’ai peur de me tromper dans les doses à prendre

J’ai peur de me tromper entre les différents produits à injecter

J’ai confiance dans mon traitement

Je pense que le traitement correspond à mon cas

Je pense que suivre l’ensemble des traitements est compliqué

Je pense que le traitement à un impact sur ma vie quotidienne

Je pense que le traitement à un impact sur ma santé

**A tous**

**Q22. D’après votre expérience personnelle et dans votre situation, considérez-vous les examens de monitoring (les échographies ains que les prises de sang) comme :**

1. Très contraignants
2. Plutôt contraignants
3. Plutôt pas contraignants
4. Pas du tout contraignants

**A tous**

**Q27. Aujourd’hui quelle note entre 1 et 10 donneriez-vous pour évaluer votre niveau de satisfaction concernant votre parcours d’AMP :**

*1 veut dire que vous êtes très insatisfait de votre parcours d’AMP. 10 veut dire que vous êtes très satisfait de votre parcours d’AMP. Les notes intermédiaires vous permettent de nuancer votre jugement*

Satisfation globale par rapport à votre parcours d’AMP : /__/__/ / 10

**A tous**

**Q28. Et quelle note entre 1 et 10 donneriez-vous pour évaluer votre niveau de satisfaction à chacune des étapes suivantes de votre parcours d’AMP. Si vous n’êtes pas ou n’avait pas été concerné par certaines de ces étapes merci de sélectionner la réponse ”non concerné”.**

*1 veut dire que vous êtes très insatisfait de votre parcours d’AMP. 10 veut dire que vous êtes très satisfait de votre parcours d’AMP. Les notes intermédiaires vous permettent de nuancer votre jugement*

1. /__/__/ / 10
2. Non concerné
3. Insémination artificielle avec sperme du conjoint (IAC)
4. Insémination artificielle avec don de sperme (IAD)
5. Fécondation *in Vitro*
6. Fécondation *in Vitro* avec don de gamètes
7. Accueil d’embryon(s)
8. Situations avec transfert d’embryon (TEC ou TEV, double don, accueil d’embryon)

**A tous**

**Q29. Sur les [insérer réponse en RS11] que vous avez réalisées, combien n’ont pas abouti ?**

1. /__/__/ tentatives qui n’ont pas abouti

**Aux personnes qui ont connu au moins 1 tentative qui n’a pas abouti**

**Q30. Et comment vous-êtes vous senti(e)s lorsqu’une tentative n’a pas abouti ?**

1. Perdu (e)
2. Rassuré (e)
3. Espoir
4. Seul (e)
5. Angoissé (e)
6. Confiant (e)
7. Différent (e)
8. Honteux (euse)
9. En colère
10. Pris (e) en charge
11. Découragé(e)
12. Résigné (e)

**Aux femmes**

**Q31. Combien de fausses couches avez-vous vécu dans le cadre de votre parcours d’AMP ? :**

*Si vous n’avez vécu aucune fausse couche merci d’indiquez 0.*

/__/__/ fausses couches dans le cadre du parcours d’AMP

**Aux femmes qui ont eu au moins 1 fausse couche**

**Q32. Quelle prise en charge vous a été proposée lors de votre dernière fausse couche :**

1. Suivi du taux d’hCG
2. Suivi échographique
3. Prescription médicamenteuse d’un médicament pour vous faire expulser
4. Traitement chirurgical par aspiration
5. Attente de l’expulsion naturelle
6. Autre : Précisez : _____________

**Aux femmes qui ont eu au moins 1 fausse couche**

**Q33. Au bout de combien de fausses couches un bilan spécifique vous a-t-il été proposé ?**

1. Dès la première
2. Dès la deuxième
3. Dès la troisième
4. Après 4 fausses couches ou plus
5. Jamais malgré des fausses couches répétées
6. Jamais car ce n’était pas utile

**L’impact psychologique**

**A tous**

**Q35. Quelle note entre 1 et 10 donneriez-vous aujourd’hui pour évaluer l’impact psychologique de votre parcours d’AMP ?** *1 signifie que vous ne ressentez aucune conséquence psychologique vis à vis de votre parcours d’AMP et 10 que vous estimez être très impacté(e) psychologiquement. Les notes intermédiaires servent à nuancer votre jugement.*

**Impact psychologique** du parcours d’AMP : /__/__/ / 10

**A tous**

Q36. Diriez-vous qu’il vous arrive… ?

1. Très souvent
2. Souvent
3. Parfois
4. Rarement
5. Jamais
6. De vous sentir stressé(e)
7. De vous sentir fatigué (e)
8. D’avoir envie de tout lâcher, de disparaître
9. D’être fier/fière de ce que vous faites
10. De vous sentir déconnecté(e) de la vie ordinaire
11. De vous sentir en échec
12. De vous sentir confiant (e)
13. D’avoir l’impression de vivre une double vie (entre ce que vous ressentez intérieurement et l’image que vous montrez à l’extérieur)
14. De vous sentir incompris(e)
15. D’avoir l’impression que la vie est injuste
16. De vous sentir discriminé (e) (socialement, amicalement, professionnellement...)

**A tous**

Q37. Et dans le cadre de votre parcours d’AMP diriez-vous que vous êtes d’accord ou pas avec chacune des affirmations suivantes :

1. Tout à fait d’accord
2. Plutôt d’accord
3. Plutôt pas d’accord
4. Pas du tout d’accord
5. Non concerné
6. Je suis anxieux/anxieuse à l’approche des rendez-vous d’AMP
7. Je suis impatient(e) d’aller à mon rendez-vous avec mon gynécologue
8. J’ai toujours peur que l’on m’annonce que je ne pourrai jamais avoir d’enfant
9. Les consultations dans le cadre de l’AMP sont bien réparties dans le temps
10. Je ne supporte plus l’attente du résultat à chaque tentative
11. Je suis sur(e) que cela va finir par marcher
12. Les rendez-vous d’AMP sont faciles d’accès en termes d’horaires
13. J’ai peur d’avoir toujours trop de questions à poser à l’équipe médicale
14. J’ai peur de craquer
15. Je suis fataliste, je fais ce que j’ai à faire

**L’impact physique**

**A tous**

**Q38. Quelle note entre 1 et 10 donneriez-vous aujourd’hui pour évaluer l’impact physique de votre parcours d’AMP ?** *1 signifie que vous ne ressentez aucune conséquences physiques vis à vis de votre parcours d’AMP et 10 que vous estimez être très impacté(e) physiquement. Les notes intermédiaires servent à nuancer votre jugement.*

**Impact physique** du parcours d’AMP : /__/__/ / 10

**A tous**

**Q39. Et dans le détail, à quel point les éléments suivants se sont-ils manifestés par rapport à votre état physique ces derniers mois :**

1. Beaucoup
2. Moyennement
3. Un peu
4. Pas du tout
5. Bonne capacité à supporter les traitements
6. Douleurs lors des ponctions ovariennes
7. Sentiment d’intense fatigue ou d’épuisement
8. Envie de dormir pendant la journée
9. Incapacité à sortir de chez vous
10. Troubles de l’humeur
11. Variations de poids (perte ou prises de poids)
12. Troubles du sommeil
13. Sentiment d’irritabilité
14. Difficultés à vous concentrer la journée
15. Perte d’appétit
16. Répercussions sur la vie sexuelle

**L’impact sur la vie affective**

**A tous**

**Q40. Quelle note entre 1 et 10 donneriez-vous aujourd’hui pour évaluer l’impact de votre parcours d’AMP sur votre vie affective ?** *1 signifie que vous ne ressentez aucune conséquences au sein de votre vie affective vis à vis de votre parcours d’AMP et 10 que vous estimez être très impacté(e) dans votre vie affective. Les notes intermédiaires servent à nuancer votre jugement.*

**Impact** du parcours d’AMP **sur la vie affective** : /__/__/ / 10

**Aux personnes en union libre, mariées ou pacsées**

**Q41. Et dans le détail, à quel point les éléments suivants se sont-ils manifestés dans votre vie de couple ces derniers mois :**

1. Beaucoup
2. Moyennement
3. Un peu
4. Pas du tout
5. Diminution du désir pour votre partenaire
6. Absence de rapports sexuels pendant plusieurs semaines voire plusieurs mois
7. Sentiment d’inégalité au sein de votre couple dans le vécu du parcours d’AMP
8. Irritabilité vis à vis de votre conjoint
9. Difficultés à avoir des rapports sexuels
10. Sentiment d’injustice vis à vis de votre conjoint
11. Tensions répétées pour des ”petits problèmes” de la vie quotidienne
12. Envie de vous séparer/de divorcer de votre conjoint
13. Volonté de vous recentrer sur votre couple
14. Un plus grand plaisir ressenti dans les ”petits moments du quotidien” avec votre conjoint
15. Soudé et combatif face à l’adversité
16. Sur la même longueur d’ondes

**Aux personnes en union libre, mariées ou pacsées**

**Q42. Et diriez-vous que pendant votre parcours d’AMP, votre relation de couple est devenue beaucoup plus, un peu plus, un peu moins, beaucoup moins ou ni plus, ni moins...**

1. Beaucoup plus
2. Un plus
3. Un peu moins
4. Beaucoup moins
5. Ni plus, ni moins
6. Forte
7. Difficile
8. Tendue
9. Complice
10. Lointaine
11. Soudée
12. Fatigante
13. Source d’incompréhension

**L’impact sur les relations avec l’entourage**

**A tous**

**Q43. Quelle note entre 1 et 10 donneriez-vous aujourd’hui pour évaluer l’impact de votre parcours d’AMP sur vos relations les personnes de votre entourage (famille, ami, collègues...) ?** *1 signifie que votre parcours d’AMP n’a aucun impact sur vos relations avec les personnes de votre entourage et 10 que vous estimez être très impacté(e) dans vos relations avec votre entourage. Les notes intermédiaires servent à nuancer votre jugement.*

**Impact** du parcours d’AMP sur les relations avec l’entourage : /__/__/ / 10

**A tous**

**Q44. Et dans le détail, à quel point les éléments suivants se sont-ils manifestés dans vos relations avec votre entourage ces derniers mois :**

1. Beaucoup
2. Moyennement
3. Un peu
4. Pas du tout
5. De vous dire que personne ne peut comprendre ce que vous vivez
6. De sentir souvent la pression de votre entourage par rapport à votre projet d’enfant
7. D’être jaloux(se) de personnes enceintes autour de vous
8. D’avoir envie de rompre une relation amicale ou familiale parce que la personne concernée était enceinte
9. De vous sentir culpabilisé(e) par votre entourage
10. De ne plus supporter le bonheur des autres autour de vous
11. De vous sentir soutenu(e) par votre famille, vos amis
12. De lire de la déception dans le regard des personnes de votre entourage par rapport à votre situation
13. De ne plus supporter les gens qui prennent constamment de vos nouvelles
14. De trouver du réconfort dans les réseaux sociaux grâce aux relations que vous pouvez y nouer

**L’impact sur la vie professionnelle**

**Aux personnes actives**

**Q45. Quelle note entre 1 et 10 donneriez-vous aujourd’hui pour évaluer l’impact de votre parcours d’AMP sur votre vie professionnelle ?** *1 signifie que votre parcours d’AMP n’a aucun impact sur votre vie professionnelle et 10 que vous estimez être très impacté(e) dans votre vie vie professionnelle. Les notes intermédiaires servent à nuancer votre jugement.*

**Impact** du parcours d’AMP sur **votre vie professionnelle** : /__/__/ / 10

**Aux personnes actives**

Q46. Personnellement, avez-vous le sentiment que votre parcours d’AMP a un impact important ou pas sur…

1. Un impact très important
2. Un impact plutôt important
3. Un impact plutôt pas important
4. Un impact pas du tout important
5. Aucun impact
6. Votre évolution en termes de responsabilités professionnelles
7. Votre évolution en termes de salaire
8. L’organisation de votre temps de travail
9. Votre niveau de stress au travail
10. Votre bien-être au travail
11. Votre projet professionnel
12. La qualité de votre travail
13. Votre motivation pour aller travailler le matin
14. Vos capacités physiques/intellectuelles pour travailler
15. Les relations socio-affectives avec vos collègues de travail

**Aux personnes actives**

Q47. Et aujourd’hui comment vous sentez-vous dans le cadre de votre travail ?

1. Très bien
2. Plutôt bien
3. Plutôt mal
4. Très mal

**Aux personnes actives**

Q48. Plus précisément, êtes-vous confronté ou avez-vous été confronté aux situations suivantes en raison de votre parcours d’AMP ?

1. Oui
2. Non
3. Réduire votre nombre d’heures de travail
4. Augmenter votre nombre d’heures de travail
5. Obtenir de votre employeur un aménagement de votre travail (horaires de travail, travail à domicile, changement d’activité…)
6. Etre licencié
7. Démissionner pour vous consacrer en totalité à votre parcours d’AMP
8. Etre sans activité professionnelle pendant un certain temps
9. Changer d’employeur
10. Prendre un ou plusieurs arrêt(s) maladie
11. Arrêter votre activité (travailleur indépendant)
12. Renoncer à une opportunité (promotion, mobilité géographique)
13. Obtenir de votre employeur des autorisations exceptionnelles d’absence
14. Utiliser les autorisations d’absence prévues par la loi pour le protocole de soins en AMP
15. Subir des pressions de votre employeurs, des vos collègues à cause de votre parcours d’AMP
16. Ressentir de la compréhension sur votre situation de la part de votre employeur
17. Mentir à votre employeur pour justifier des absences

**L’impact financier**

**A tous**

**Q49. Quelle note entre 1 et 10 donneriez-vous aujourd’hui pour évaluer l’impact financier de votre parcours d’AMP ?** *1 signifie que votre parcours d’AMP n’a aucune conséquence financière et 10 que vous estimez être très impacté(e) financièrement. Les notes intermédiaires servent à nuancer votre jugement.*

**Impact financier** du parcours d’AMP : /__/__/ / 10

**A tous**

**Q50. Avez-vous déjà été confronté aux situations suivantes du fait de votre parcours d’AMP ?**

1. Jamais
2. Rarement
3. Quelquefois
4. Souvent
5. Très souvent
6. Cacher à vos proches les problèmes financiers que vous pouvez rencontrer du fait de votre parcours d’AMP
7. Emprunter de l’argent à votre entourage pour « réussir à joindre les deux bouts » à cause des dépenses liées à l’AMP
8. Etre obligé(e) de puiser dans vos réserves financières pour pouvoir vous en sortir
9. Rencontrer des difficultés pour obtenir la prise en charge à 100% du fait d’être dans un parcours d’AMP
10. Etre remboursé(e) tardivement après le moment où vous avez dû avancer certains frais dans le cadre de votre parcours d’AMP
11. Manquer d’accompagnement par rapport aux démarches administratives à réaliser lorsque l’on débute un parcours d’AMP
12. Ne pas obtenir de réponses à vos questions de la part de l’Assurance Maladie ou de votre mutuelle concernant la prise en charge financière d’un parcours d’AMP
13. Ouvrir une cagnote en ligne pour récolter les fonds nécessaires à votre prochain protocole de soins en AMP
14. Financer des examens et/ou utiliser des dispositifs médicaux d’AMP non remboursés par la Sécurité Sociale

**L’impact sur les projets de vie**

**A tous**

**Q51. Quelle note entre 1 et 10 donneriez-vous aujourd’hui pour évaluer l’impact de votre parcours d’AMP sur vos projets de vie ?** *1 signifie que votre parcours d’AMP n’a aucune conséquence sur vos projets de vie et 10 que vous estimez être très impacté(e) pour vos projets de vie futurs. Les notes intermédiaires servent à nuancer votre jugement.*

**Impact** du parcours d’AMP **sur vos projets de vie** : /__/__/ / 10

**A tous**

**Q52. Votre parcours d’AMP vous a-t-il amené à anticiper, concrétiser, retarder ou renoncer à chacun des projets suivants ?**

1. Anticiper
2. Concrétiser
3. Retarder
4. Renoncer
5. Non concerné(e)
6. Demander un prêt pour un achat immobilier
7. Voyager
8. Vous marier/pacser
9. Divorcer, vous séparer de votre conjoint(e)
10. Adopter un enfant
11. Devenir bénévole dans une association
12. Déménager
13. Questionner votre réel désir d’être parent
14. Faire une pause dans le cadre de votre parcours d’AMP
15. Aborder la question de l’avenir avec votre conjoint/compagnon
16. Changer de travail ou de poste
17. Faire une formation professionnelle

**L’impact sur les relations avec les professionnels de santé**

**A tous**

**Q53. Les relations que vous entretenez avec les professionels de santé suivant dans le cadre de votre parcours d’AMP sont-elles très bonnes, plutôt bonnes, assez mauvaises, très mauvaises ?**

1. Très bonnes
2. Plutôt bonnes
3. Assez mauvaises
4. Très mauvaises
5. Je n’ai pas de contact avec ce professionnel de santé
6. Le gynécologue qui vous suit/suit votre conjointe/compagne habituellement
7. Le médecin spécialisé en AMP
8. Votre médecin généraliste
9. L’infirmière à domicile
10. Le psychologue du centre d’AMP
11. Le biologiste du centre d’AMP
12. Les sages-femmes ou infirmières du centre d’AMP
13. Le pharmacien

**A tous**

**Q54. Avez-vous le sentiment que les professionnels de santé impliqués dans votre parcours d’AMP font les choses suivantes ?**

1. Oui tout à fait
2. Oui plutôt
3. Non plutôt pas
4. Non pas du tout
5. Planifier suffisamment à l’avance les différents examens à réaliser
6. Vous considérer comme un acteur à part entière de votre parcours d’AMP
7. Vous délivrer un niveau d’information satisfaisant sur le diagnostic, les examens, les traitements et leurs effets secondaires,
8. Vous écouter et vous accompagner lorsque vous exprimez des difficultés dans le cadre de votre parcours d’AMP
9. Etre disponible pour vous conseiller sur ce que vous pouvez faire pour mieux vivre votre parcours d’AMP

**Partie 4: Attentes et besoins**

**A tous**

**Q55. Pour chacun des éléments suivants, pensez-vous que vous avez toutes les informations dont vous avez besoin ?**

1. Oui tout à fait
2. Oui plutôt
3. Non plutôt pas
4. Non pas du tout
5. Sur l’ensemble des techniques d’AMP disponibles en France
6. Sur l’ensemble des techniques d’AMP disponibles à l’étranger
7. Sur les différentes étapes d’un parcours d’AMP
8. Sur les modes de prise en charge dans le cadre d’une AMP (stimulation, insémination, FIV...)
9. Sur les aides (psychologiques, assistante sociale, associations de patients...) dont vous pouvez bénéficier dans le cadre d’un parcours d’AMP
10. Sur les conditions d’éligibilité à un parcours d’AMP (conditions administratives, médicales, choix des dossiers selon les centres...)
11. Sur la nombre de tentatives d’AMP que vous pouvez réaliser en France (tentatives remboursées, décompte d’une tentative même en cas de fausse couche…)
12. Sur les traitements aujourd’hui disponibles (la fréquence de prise, la conservation des produits, le mode d’injection, les effets...)
13. Sur l’impact des traitements sur la santé des femmes
14. Sur l’impact des traitements sur la santé des enfants
