# Supplementary material for: Psychosocial and professional burden of Medically Assisted Reproduction (MAR): Results from a French survey
Source: PLoS One. 2020 Sep 24;15(9):e0238945. doi: 10.1371/journal.pone.0238945 (PMC7514013; doi:10.1371/journal.pone.0238945)
Supplement: S2 File — (DOCX) [file pone.0238945.s002.docx]

**Part 1: Demographic questions**

**For all**

**RS1. You are:**

1. A man
2. A woman

**For all**

**RS2. What is your date of birth?**

**I__I__I Month**

**I__I__I__I__I Year**

1. **STOP HERE if you are < 18 or > 50 years old**

**For all**

**Q0. On a scale of 1 to 10, what score would you give yourself today concerning your general wellbeing?** *1 being that you do not feel well, and 10 that you feel very well. Intermediate scores help to qualify your judgment.*

1. Level of general wellbeing: /__/__/ / 10

**If you have children**

**RS3. Among your children, have you had any of them using medically assisted reproduction (MAR)?** As a reminder, MAR are a set of clinical and biological practices where medicine intervenes more or less directly in procreation to conceive a child.

1. Yes
2. No 🡺 **STOP**

**For all**

**RS4. What is your family situation?**

1. Single

2. Common-law (couple status)

3. Married

4. Separated

5. Divorced

6. Widowed

7. Pacsé (e) (Civil Solidarity Pact).

**For couples**

**RS5. Are you currently undergoing or have you undergone medically assisted reproduction (MAR)?**

1. Yes
2. No 🡺 **STOP**

**For women**

**RS6. Are you currently pregnant?**

1. Yes
2. No

**For pregnant women**

**RS7. Are you pregnant after having undergone medically assisted reproduction (MAR)?**

1. Yes
2. No

**For subjects without children:**

**RS8. Have you undergone medically assisted reproduction (MAR) in your lifetime to conceive a child?**

1. Yes
2. No 🡺 **STOP**

**For all**

**RS9. Where do you find yourself today with regards to the MAR process?**

1. I am currently undergoing MAR in France

2. I am currently undergoing MAR abroad 🡺 **STOP**

3. I finished MAR care after becoming a parent thanks to MAR carried out abroad 🡺 **STOP**

4. I finished MAR care because I am expecting a child thanks to MAR carried out abroad 🡺 **STOP**

5. I finished MAR care because I am a parent

6. I finished MAR care because I am expecting a child

7. I stopped MAR care for personal reasons

8. I stopped MAR care because it is no longer covered by social healthcare

9. I stopped MAR care because the doctors no longer want to take care of me for medical reasons

10. I stopped MAR care because of too many failures

11. I am taking a break from MAR healthcare

12. I am waiting to start MAR care because I am on a waiting list

**If currently undergoing MAR (RS9=1)**

**RS10. What type of MAR protocol are you undergoing?**

1. Intrauterine insemination with partner’s sperm

2. Intrauterine insemination with donor sperm

3. In-Vitro Fertilization

4. In-Vitro fertilization with donor sperm or donor oocytes

5. Embryo donation program

6. Embryo transfer (in the case of frozen embryo transfers, or transfer of donor embryos)

**For all**

**RS11. How many MAR processes have you undergone in your life?**

*By MAR processes we mean any MAR protocol begun, in which you were followed by a doctor specialized in MAR and during which you benefited from different types of medical care such as intrauterine inseminations and IVF until you or the doctor decides to stop the MAR or you are pregnant.*

I__I__I MAR processes

**For all**

**RS11. And how many cycles have you undergone within your MAR process(es)?**

I__I__I cycles

**Part 2: Healthcare history before MAR**

**For all**

**Q1. Among the following, which initiated your consideration of getting MAR care?**

1. Following a consultation with your general practitioner

2. Following a consultation with your gynaecologist

3. A discussion with people close to you

4. Information you found on the internet

5. Blogs, forums on assisted reproduction

6. Informational campaigns on television, the radio, or the internet

7. Several months or several years without successfully conceiving a child

**For all**

**Q2. How long have you been trying to have a baby?**

**I__I__I Month**

**I__I__I__I__I Year**

**For all**

**Q3. When you encountered these first difficulties, what were all the questions you asked yourself at that time?**

1. Is it my fault? 2. Is it my spouse’s / partner’s fault? 3. Is it hereditary? 4. Is there a history in my family or that of my spouse’s? 5. Will I have to adopt? 6. Will I ever have a child? 7. Will I be eligible for an MAR? 8. Is it related to my diet? 9. Is it related to my weight? 10. Is it related to my lifestyle (smoking, physical activity, alcohol ...) 11. Is it related to my professional environment?

**For all**

**Q4. Who did you meet first to talk about your difficulties in conceiving a child?**

1. Your / your spouse’s / partner’s gynaecologist

2. A doctor specialized in MAR

3. Your general practitioner

4. An endocrinologist

5. A midwife

6. A member of my family / those close to me (mother, sister, friend)

7. Other: Specify: ____________________________

**For all**

**Q5. Who diagnosed your difficulties in conceiving a child?**

1. Your gynaecologist

2. A gynaecologist specialized in MAR

3. Your general practitioner

**For all**

**Q11. When did you consult regarding your project to have a child?**

1. After less than 6 months of trying 2. Between 6 months and 1 year of trying 3. Between 1 year and 18 months of trying 4. Between 18 months and 24 months of trying 5. After 24 months of trying

**For all**

**Q8. When did the first consultation with the MAR specialist take place?**

**I__I__I Month**

**I__I__I__I__I Year**

**For all**

**Q10. Among the following feelings, what are the 3 that you felt during the first consultation with the doctor specialized in MAR?**

1. Lost

2. Reassured

3. Hope

4. Alone

5. Anxious

6. Confident

7. Different

8. Ashamed

9. Angry

10. Taken care of

11. Discouraged

12. Resigned

**For all**

**Q12. As part of your MAR care, what advice or recommendations were offered to you when you were diagnosed with your difficulties in conceiving a child?**

1. A change in diet

2. The practice of regular physical activity

3. Weight loss

4. Stop / reduce smoking

5. Stop / decrease cannabis consumption

6. Stop / decrease alcohol consumption

7. Surgical intervention

8. A complete infertility evaluation for the couple for quick and active MAR management in sight

9. Nothing, just keep trying and come back in a while

0. Other: Specify: ___________________

**Part 3: MAR care**

*Introduction: we are now going to come back to the MAR care that you are undergoing or that you have already undergone in your life. If you have ever undergone several MAR processes, we ask you to answer the following questions by considering the last MAR process for which you were cared for by a doctor specialized in MAR and during which you were able to benefit from different types of MAR such as intrauterine insemination and IVF until you or the doctor decided to stop the MAR or you are/were pregnant.*

**If currently undergoing MAR procedures or if you underwent a procedure without having a child**

**Q13. How often do you think about your desire to have children today?**

1. All the time

2. Several times a day

3. At least once a day

4. Several times a week

5. At least once a week

6. At least once a month

7. Seldom

8. Never

**For all**

**Q14. When did your first MAR consultation take place?**

**I__I__I Month**

**I__I__I__I__I Year**

**For all**

**Q15. What were the main feelings you felt during your first MAR consultation?**

1. Lost

2. Reassured

3. Hope

4. Alone

5. Anxious

6. Confident

7. Different

8. Ashamed

9. Angry

10. Taken care of

11. Discouraged

12. Resigned

**For all**

**Q16. On average, how long does it take you to get to your MAR centre regardless of the mode of transportation you use?**

1. Less than 15 min

2. Between 15 and 30 min

3. Between 30 min and 1 hour

4. Between 1h and 2h

5. More than 2h

**For all**

**Q17. Would you say that you were very, somewhat, somewhat not or not at all satisfied with the following as part of your MAR care?**

1. Very satisfied

2. Somewhat satisfied

3. Somewhat dissatisfied

4. Not at all satisfied

1. Overall reception (staff, facilities ...) upon entering the MAR protocol

2. Explanations provided by the medical staff (on MAR care, on the reasons for your difficulties in having a child ...)

3. The quality of care provided at the MAR centre

4. The involvement of medical staff in your MAR journey

5. Availability of medical staff to answer your questions during the treatment protocol

6. Non-medical support offered (psychologist, patient organisations, etc.)

7. The average length of consultation for your MAR course

8. The ability of medical staff to answer your questions

9. The ability of medical staff to reassure you

**For all**

**Q18. As part of your MAR journey how often do you visit forums, social networks...?**

1. Very often

2. Often

3. Rarely

4. Never

**To those who visit social networks**

**Q19. Why do you visit forums, social networks / forums as part of your MAR care?**

1. To find out about the different steps of the MAR

2. To share your experience

3. To have support throughout the process, to seek comfort

4. To interact with people who understand you

5. To express yourself freely

6. To share your story

7. To say what you cannot say to your spouse / partner, the people close to you or the medical staff

8. To find answers to questions you ask about your personal journey

**Q20. Have you already contacted one or more organization (s)?**

1. Yes

2. No

**For all**

**Q21. Indicate for each of the following adjectives whether it applies yes or no to the way you perceived the diagnostic evaluation carried out at the start of your MAR care:**

1 Yes

2 No

1. Cumbersome

2. Useful

3. Reassuring

4. Necessary

5. Confusing

6. Long

7. Incomprehensible

8. Worrisome

To people currently or formerly undergoing ovarian stimulation by injections

**Q21BIS. As part of your ovarian stimulation treatment, would you say that you agree or disagree with each of the following statements?**

1. Strongly agree

2. Somewhat agree

3. Somewhat disagree

4. Strongly disagree

5. Not concerned

1. I understand the prescribed treatment

2. I feel comfortable with self-injections

3. I get help from a nurse or someone close for the injections

4. I am afraid of getting the doses wrong

5. I'm afraid of making a mistake between the different products to be injected

6. I am confident in my treatment

7. I think the treatment is right for me

8. I think that going through all of the treatments is complicated

9. I think the treatment has an impact on my daily life

10. I think the treatment has an impact on my health

**For all**

**Q22. From your personal experience and in your situation, you consider monitoring exams (ultrasounds as well as blood tests) as:**

1. Very cumbersome

2. Rather cumbersome

3. Rather not cumbersome

4. Not at all cumbersome

**For all**

**Q27. Today, what score between 1 and 10 would you give to assess your level of satisfaction with your MAR care?**

*1 being that you are very dissatisfied with your MAR care. 10 being that you are very satisfied with your MAR care. Intermediate scores allow you to qualify your judgment.*

Overall satisfaction with your MAR care: /__/__/ / 10

**For all**

**Q28. And what score between 1 and 10 would you give to assess your level of satisfaction at each of the following stages of your MAR care. If you are not or have not been affected by some of these steps, please select the answer "not concerned".**

*1 being that you are very dissatisfied with your MAR care. 10 being that you are very satisfied with your MAR care. Intermediate scores allow you to qualify your judgment.*

1. / __ / __ / / 10

2. Not concerned

1. Intrauterine insemination with partner sperm

2. Intrauterine insemination with donor sperm

3. In-Vitro Fertilization

4. In-Vitro fertilization with donor sperm

5. Embryo donation program

6. Embryo transfer (in the case of frozen embryo transfers, or transfer of donor embryos)

**For all**

**Q29. Of the [insert RS11 response] that you made, how many failed?**

1. / __ / __ / unsuccessful attempts/processes?

**To people who have experienced at least 1 unsuccessful attempt/process**

**Q30. And how did you feel when an MAR process was unsuccessful?**

1. Lost

2. Reassured

3. Hopeful

4. Alone

5. Anxious

6. Confident

7. Different

8. Ashamed

9. Angry

10. Taken care of

11. Discouraged

12. Resigned

**For women**

**Q31. How many miscarriages have you experienced in MAR?**

*If you have not experienced any miscarriages please enter 0.*

/ __ / __ / miscarriages in MAR

**For women who have had at least 1 miscarriage**

**Q32. What treatment was offered to you during your last miscarriage?**

1. Monitoring the hCG level

2. Ultrasound monitoring

3. Prescription of a drug to expel the foetus

4. Surgical aspiration treatment

5. Waiting for natural expulsion

6. Other: Specify: _____________

**For women who have had at least 1 miscarriage**

**Q33. After how many miscarriages was a specific assessment offered to you?**

1. From the first 2. From the second 3. From the third 4. After 4 or more miscarriages 5. Never despite repeated miscarriages 6. Never because it was not useful

**Psychological impact**

**For all**

**Q35. What score between 1 and 10 would you give today to assess the psychological impact of your MAR care? 1 being that you do not feel any psychological consequences with regard to your MAR journey and 10 means that you consider yourself to be very psychologically impacted. The intermediate scores allow you to qualify your judgment.**

**Psychological impact of MAR care:**/__/__/ / 10

**For all**

**Q36. Would you say it happens to you...?**

1. Very often

2. Often

3. Sometimes

4. Rarely

5. Never

1. To feel stressed

2. To feel tired

3. To want to stop everything, to disappear

4. To be proud of what you do

5. To feel disconnected from ordinary life

6. To feel like a failure

7. To feel confident

8. To have the impression of living a double life (between what you feel inside and the image you portray on the outside)

9. To feel not understood

10. Feel like life is unfair

11. To feel discriminated against (socially, friend-wise, professionally ...)

**For all**

**Q37. And as part of your MAR journey would you say that you agree or disagree with each of the following statements:**

1. Strongly agree

2. Somewhat agree

3. Somewhat disagree

4. Strongly disagree

5. Not concerned

1. I am increasingly anxious as my MAR appointments get closer

2. I am impatient to go to my appointment with my gynaecologist

3. I am always afraid that it will be announced that I can never have children

4. Consultations within the framework of MAR care are well distributed over time

5. I can't stand waiting for the results any more after each MAR attempt

6. I'm sure it will end up working

7. MAR appointments are easy to schedule

8. I'm afraid I always have too many questions to ask the medical team

9. I'm afraid I will break down

10. I am a fatalist, I do what I have to do

**Physical impact**

**For all**

**Q38. What score between 1 and 10 would you give today to assess the physical impact of your MAR care***? 1 means that you do not feel any physical consequences with regards to your MAR care and 10 means that you consider yourself to be very physically impacted. The intermediate scores serve to qualify your judgment.*

**Physical impact** of the MAR care**: / __ / __ / / 10**

**For all**

**Q39. In detail, how much have you experienced in relation to your physical condition in recent months:**

1. Lots

2. Moderately

3. Little

4. Not at all

1. Good ability to tolerate treatments 2. Pain during oocyte retrieval 3. Feeling of intense fatigue or exhaustion 4. Wanting to sleep during the day 5. Inability to leave your home 6. Mood disorders 7. Weight changes (weight loss or gain) 8. Sleep disturbances 9. Feeling irritable

10. Concentration difficulties during the day

11. Loss of appetite

12. Consequences on sexual life.

**The impact on emotional life**

**For all**

**Q40. What score between 1 and 10 would you give today to assess the impact of your MAR care on your emotional life?** *1 being that you do not feel any consequences on your emotional life with regards to your MAR care and 10 that you consider to be very emotionally impacted. The intermediate scores serve to qualify your judgment.*

**Impact of MAR on emotional life:** / __ / __ / / 10

**To couples**

**Q41. In detail, to what extent have you experienced the following in your relationship in recent months?**

1. Lots

2. Moderately

3. A little

4. Not at all

1. Decreased desire for your partner

2. Absence of sexual intercourse for several weeks or even months

3. Feeling of inequality within your couple in how MAR was experienced

4. Irritability towards your partner

5. Difficulties having sex

6. Feeling of injustice towards your partner

7. Repeated tensions for "small problems" of daily life

8. Wanting to separate / divorce your partner/spouse

9. Willingness to refocus on your relationship

10. A greater pleasure felt in the “little moments of daily life” with your partner

11. United and powerful the face of adversity

12. On the same wavelength

**For couples**

**Q42. Would you say that during your MAR care, your relationship as a couple has become much more, a little more, a little less, a lot less or neither more nor less...?**

1. Much more

2. A little more

3. A little less

4. A lot less

5. Neither more, nor less

1. Strong

2. Difficult

3. Tense

4. Accomplice

5. Divided

6. Welded

7. Tiring

8. Source of misunderstanding

**The impact on relationships with those around you**

**For all**

**Q43. What score between 1 and 10 would you give today to assess the impact of your MAR care on your relationships with people around you (family, friends, colleagues ...)?** *1 being that MAR has had no impact on your relationships with people around you and 10 being that you feel that you are very impacted in your relationships with those around you. The intermediate scores serve to qualify your judgment.*

**Impact** of MAR on relationships with those around you: / __ / __ / / 10

**For all**

**Q44. In detail, to what extent have you experienced the following in your relationship with those around you in recent months?**

1. Lots

2. Moderately

3. A little

4. Not at all

1. To tell yourself that no one can understand what you are going through

2. To often feel the pressure of those around you regarding your plans to have a child

3. Being jealous of pregnant women around you

4. To want to end a friendly or family relationship because that person was pregnant

5. To feel guilt by those around you

6. To no longer bear the happiness of others around you

7. To feel support by your family, friends

8. To feel the disappointment in the eyes of people around you regarding your situation

9. No longer tolerating people who constantly want news of you

10. Find comfort in social networks thanks to the relationships you can build there

**The impact on professional life**

**For those who work**

**Q45. What score between 1 and 10 would you give today to assess the impact of MAR on your professional life?** *1 being that MAR has had no impact on your professional life and 10 being that you feel that your professional life is very impacted. The intermediate scores serve to qualify your judgment.*

**Impact** of MAR on your professional life: / __ / __ / / 10

**For those who work**

**Q46. Personally, do you feel that MAR has had a significant impact or not on ...**

1. A very significant impact

2. A rather significant impact

3. A rather not very important impact

4. Not at all important impact

5. No impact

1. Your evolution in terms of professional responsibilities

2. Your progression in terms of salary

3. The organization of your work time

4. Your stress level at work

5. Your well-being at work

6. Your professional project/plans

7. The quality of your work

8. Your motivation to go to work in the morning

9. Your physical / intellectual capacities to work

10. Relationships with your work colleagues

**For those who work**

**Q47. How do you feel about your work environment today?**

1. Very good

2. Pretty good

3. Rather badly

4. Very badly

**For those who work**

Q48. More specifically, do you face or have you been faced with the following situations because of you are undergoing/underwent MAR?

1. Yes 2. No

1. Having to reduce your working hours

2. Having to increase your working hours

3. Having to ask your employer to rearrange work options (working hours, work at home, change of activity ...)

4. Losing your job

5. Having resigned to devote yourself entirely to your MAR care

6. Being out of work for a period of time 7. Changing jobs 8. Taking one or more sick leave (s) 9. Stopping your professional activity (self-employed) 10. Giving up an opportunity (promotion, geographic mobility) 11. Obtaining exceptional leave of absence from your employer 12. Using the leave of absence provided by law for the MAR care protocol 13. Being pressured by your employers, your colleagues because you are undergoing MAR

14. Feeling understanding of your situation by your employer 15. Lying to your employer to justify absences

**Financial impact**

**For all**

**Q49. What score between 1 and 10 would you give today to assess the financial impact of MAR care on you?** *1 being that MAR has had no financial consequences and 10 meaning that it has had a significant financial impact. The intermediate scores serve to qualify your judgment.*

**Financial impact of MAR: / __ / __ / / 10**

**For all**

**Q50. Have you ever faced the following situations because you underwent/are undergoing MAR?**

1. Never

2. Rarely

3. Sometimes

4. Often

5. Very often

1. Hiding from your loved ones the financial problems you may encounter as a result of MAR care.

2. Borrowing money from those around you to "make ends meet" because of MAR expenses

3. Being forced to draw on your financial reserves

4. Encountering difficulties in obtaining 100% government medical care support for MAR

5. Being reimbursed late and having to advance certain costs to pay for MAR

6. Lacking support in the administrative procedures needed to be carried out when one begins MAR care

7. Not getting answers to your questions from government medical care support or Health Insurance concerning the financial coverage of MAR

8. Starting an online fund raising for costs of your next MAR treatment protocol

9. Paying for exams and / or medical devices for MAR that are not reimbursed by the government medical care support system.

**Impact on life projects**

**For all**

**Q51. What score between 1 and 10 would you give today to assess the impact of MAR on your life plans?** *1 being that MAR has had no impact on your life plans and 10 being that you consider that you will be very impacted for your future life plans. The intermediate scores serve to qualify your judgment.*

**Impact of MAR on your life plans: / __ / __ / / 10**

**For all**

**Q52. Did your MAR care lead you to anticipate, carry out, delay or give up on each of the following projects?**

1. Anticipate

2. Carry out

3. Delay

4. Renounce

5. Not concerned

1. Request a loan for a real estate purchase

2. Traveling

3. Getting married

4. Divorce, separate from your partner

5. Adopt a child

6. Become a volunteer in an organization

7. Moving

8. Question your real desire to be a parent

9. Take a break from MAR

10. Talking about the future with your spouse / partner

11. Change jobs or job position

12. Undergo professional training

**Impact on relationships with healthcare professionals**

**For all**

**Q53. Are the relationships you have with the following healthcare professionals as part of your MAR care very good, fairly good, fairly bad, very bad?**

1. Very good

2. Fairly good

3. Fairly bad

4. Very bad

5. I have no contact with this healthcare professional

1. The gynaecologist who usually treats you / your spouse / your partner

2. The doctor specialized in MAR

3. Your general practitioner

4. The home nurse

5. The MAR centre psychologist

6. The MAR centre embryologist

7. Midwives or nurses at the MAR centre

8. The pharmacist

**For all**

**Q54. Do you feel that the healthcare professionals involved in your MAR care are doing the following things?**

1. Yes, absolutely

2. Yes, rather

3. No rather not

4. Not at all

1. Plan sufficiently in advance the various exams to be carried out

2. Consider yourself as a full player in your MAR care

3. Provide you with a satisfactory level of information on the diagnosis, exams, treatments and their side effects,

4. Listen and accompany you when you express difficulties as part of your MAR treatment

5. Be available to advise you on what you can do to better live your MAR process

**Part 4: Expectations and needs**

**For all**

**Q55. For each of the following, do you think you have all the information you need?**

1. Yes, absolutely

1. Yes, rather

2. No rather not

3. Not at all

1. For all the MAR procedures available in France

2. For all the MAR procedures available abroad

3. On the different steps of an MAR process

4. On the healthcare for each MAR (stimulation, insemination, IVF ...)

5. On the help (psychological, social worker, patient organizations ...) that you can benefit from within the framework of an MAR process

6. On the conditions of eligibility for MAR (administrative, medical conditions, specific patient file requirements for each centre ...)

7. On the number of MAR attempts you can undergo in France (reimbursed attempts, counting of an attempt even in the event of miscarriage ...)

8. On the treatments available today (frequency of injections, conservation of products, mode of injection, the effects ...)

9. On the impact of treatments on women's health

10. On the impact of treatments on children's health
